# Superresolution fluorescence microscopy for 3D reconstruction of thick samples

**DOI:** 10.1186/s13041-018-0361-z

**Published:** 2018-03-15

**Authors:** Sangjun Park, Wooyoung Kang, Yeong-Dae Kwon, Jaehoon Shim, Siyong Kim, Bong-Kiun Kaang, Sungchul Hohng

**Affiliations:** 1 Department of Physics and Astronomy, Seoul National University, Seoul, Republic of Korea; 2 Institute of Applied Physics, Seoul National University, Seoul, Republic of Korea; 3 National Center of Creative Research Initiatives, Seoul National University, Seoul, Republic of Korea; 4 Research Institute of Basic Sciences, Seoul National University, Seoul, Republic of Korea; 5 School of Biological Sciences, Seoul National University, Seoul, South Korea

**Keywords:** Line-scan confocal microscopy, DNA-PAINT, Superresolution microscopy, Single-molecule localization microscopy, Three-dimensional reconstruction

## Abstract

Three-dimensional (3D) reconstruction of thick samples using superresolution fluorescence microscopy remains challenging due to high level of background noise and fast photobleaching of fluorescence probes. We develop superresolution fluorescence microscopy that can reconstruct 3D structures of thick samples with both high localization accuracy and no photobleaching problem. The background noise is reduced by optically sectioning the sample using line-scan confocal microscopy, and the photobleaching problem is overcome by using the DNA-PAINT (Point Accumulation for Imaging in Nanoscale Topography). As demonstrations, we take 3D superresolution images of microtubules of a whole cell, and two-color 3D images of microtubules and mitochondria. We also present superresolution images of chemical synapse of a mouse brain section at different z-positions ranging from 0 μm to 100 μm.

**Electronic supplementary material:**

The online version of this article (10.1186/s13041-018-0361-z) contains supplementary material, which is available to authorized users.

## Introduction

Superresolution fluorescence microscopy has made possible a variety of new discoveries previously unattainable by using conventional optical microscopes [1–14]. Current application of superresolution fluorescence microscopy to 3D reconstruction of specimens, however, is limited to thin samples. If we can apply superresolution fluorescence microscopy to reconstruct 3D structures of thick tissue samples, it will revolutionize biological studies including studies of organism developments and neural connectomics. Two main barriers need to be overcome for superresolution fluorescence microscopy to be successfully used for optical 3D reconstruction of thick biological samples. The first problem is the high level of background noise contaminating the single molecule images, which consists of strong auto-fluorescence from the bulky sample and the probe signals emitted from outside the imaging volume. The second problem is photobleaching of probes that takes place during the imaging, especially the ones lying outside the imaging volume that needs to be imaged in later processes.

Various ways have been suggested to solve the high background noise problem. HILO (Highly Inclined and Laminated Optical sheet) microscopy is successfully used to image samples with a few micron thickness, but not thicker samples [15]. SPIM (Selective Plane Illumination Microscopy) can image much thicker samples [16–23], and has a potential to reconstruct whole 3D structures of thick samples with superresolution, but it is not realized yet probably due to the complicated geometry of the setup or low collection efficiencies of the microscopes. We recently reported a video-rate line-scan confocal microscopy that can image single-molecules in thick samples with high detection efficiency [24]. However, superresolution 3D reconstruction of thick samples using the line-scan confocal microscope was not possible due to fast photobleaching of fluorescence probes outside the imaging volume.

The photobleaching problem that limits the application of superresolution fluorescence microscopy is solved by the recent introduction of DNA-PAINT method [25–32]. In this technique, a target structure is stained with short oligonucleotides called the ‘docking’ strand, and a complementary oligonucleotides labeled with a fluorescent probe (‘imager’ strand) is added into the imaging buffer. The transient bindings between the docking-imager pairs produce fluorescence blinking, which is used for single-molecule localization. Since photobleached probes are continuously replaced with a new one, fluorescence imaging can be performed without time limit. The imaging time of DNA-PAINT is inversely proportional to the ‘imager’ concentration, and it is better to use higher imager concentration for high speed imaging of DNA-PAINT. In current DNA-PAINT technology, however, the imager concentration cannot be increased much due to high background noise coming from imager strands diffusing around the docking strand, and therefore DNA-PAINT has not been used for superresolution 3D reconstruction of thick samples yet.

In this study, we combined line-scan confocal microscopy and DNA-PAINT, and successfully developed superresolution fluorescence microscopy that can image 100 μm-thick samples with both high localization accuracy and no photobleaching problem.

## Results

### Scheme of the microscope

**Fig. 1:**
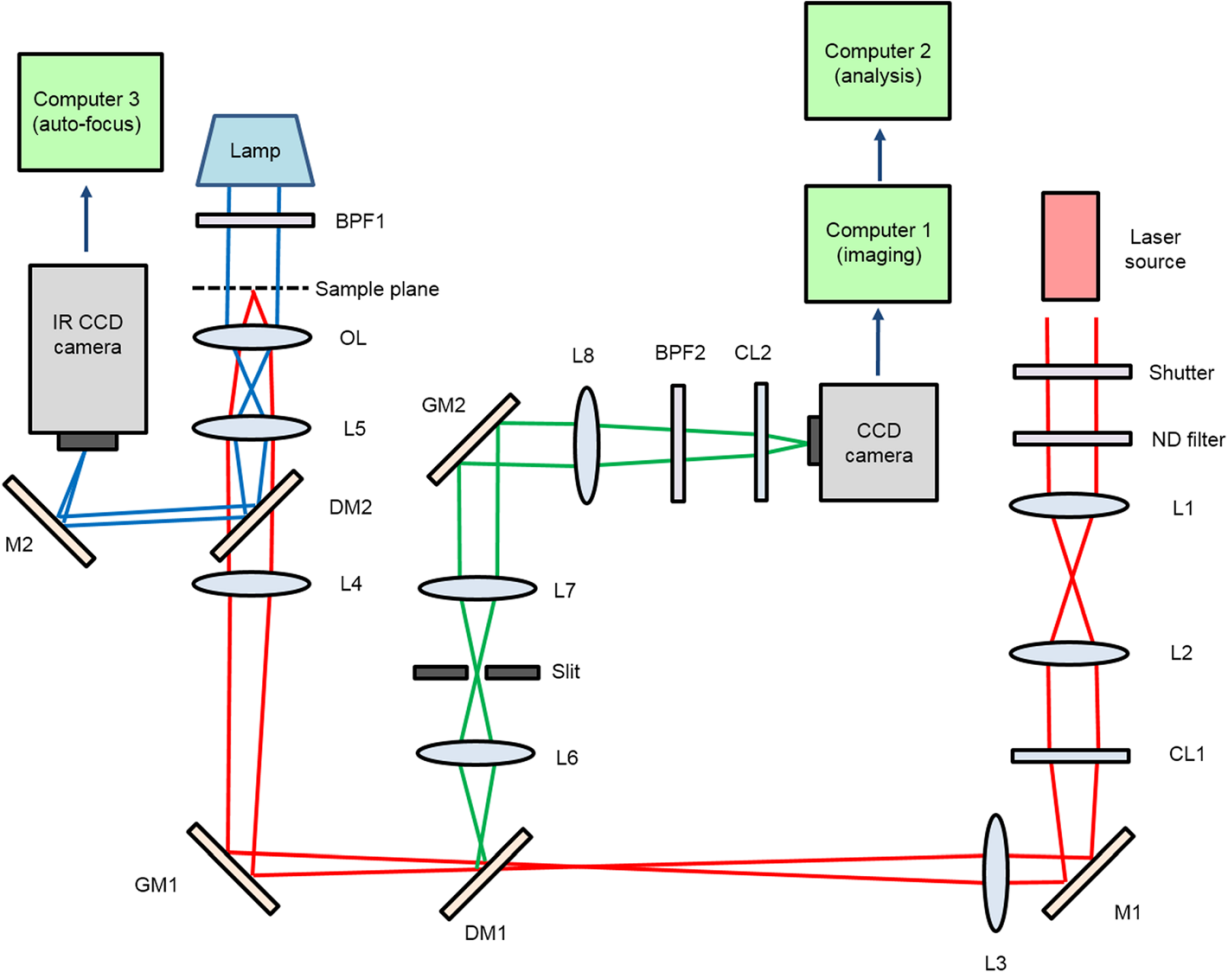
Schematic diagram of the microscope. L: lens, CL: cylindrical lens, OL: objective lens, M: mirror, DM: dichroic mirror, GM: galvanometric mirror, BPF: band pass filter

The microscope was built based on the line-scan confocal microscope that we previously reported (Fig. 1) [24]. In brief, the backport of the microscope was used to deliver both the excitation beam from a laser to a sample and the fluorescence signal from the sample to an electron multiplying-charge coupled device (EM-CCD) camera. A galvanometric mirror (GM1) was used to scan the line-focused illumination across the sample plane. The line-shaped fluorescent signal collected by the objective was projected on a confocal slit and imaged on the EM-CCD camera. To make a two-dimensional (2D) image on the EM-CCD camera, the fluorescence signal on the EM-CCD camera was scanned synchronously with GM1 by using another galvanometric mirror (GM2). Since the scanning frequency of galvanometric mirrors was 15 times larger than the image integration frequency of the EM-CCD camera, 2D images can be directly formed on the EM-CCD camera without any further data processing. During long time imaging, imaging buffer in the detection chamber was exchanged with a fresh one every hour using a syringe pump. Computer 1 controlled all the components of the EM-CCD camera, galvanometric mirrors, and syringe pump. For high speed image processing, we used an independent computer (computer 2) equipped with four graphics processing units (GPUs). To reconstruct 3D images, the z-position of fluorescence spots was determined based on optical astigmatism (Methods). Since the 3D imaging of thick samples takes quite enormous time, sample drift becomes a serious problem that should be solved. We adopted the image correlation method (Methods) for drift correction, which was controlled by using an independent computer (computer 3).

To characterize the microscope, we immobilized the docking strand (Additional file 1: Supplementary Table S1, Docking P1) and injected the imager strand (Additional file 1: Supplementary Table S1, Imager P1) labeled with Alexa Fluor 647, and binding events of the imager strand were imaged using the microscope. We confirmed that the insertion of a slit negligibly reduced the detected photon number (Additional file 1: Figure S1). To compare the z-positioning capability of the confocal microscopy and HILO microscopy, we imaged single-molecules by changing the z-position of the sample with a 100 nm step. When an oil-immersion objective was used, the x/y-width variations of fitted Gaussian function in the confocal microscopy was similar to those of HILO microscopy except that the imaging range of the confocal microscopy was reduced a little bit (Additional file 1: Figure S2a-b). When we used a water-immersion objective, the variations of x/y-width were reduced a little bit compared to those of the oil-immersion objective. To characterize the localization precision in the xy-plane, we used the estimation method reported in Thomson et al.’s paper [33]. Under the imaging condition that we used, the average detected photon number of single-molecule spots was 3730 for the oil-immersion objective and 2138 for the water-immersion objective, and thus the localization precision in the xy-plane was estimated as 5.63 nm and 8.22 nm, respectively. To characterize the localization uncertainty, we measured the standard deviation of the position histograms of surface-immobilized single-molecules using an oil-immersion objective (Additional file 1: Figure S3) [8]. When the average photon number was 623, the localization uncertainties in the x-, y-, and z-direction were 27.6 nm, 17.1 nm, and 88.0 nm, respectively. With increase average photon number (3730), the localization uncertainty in the z-direction was improved to 29.4 nm (Additional file 1: Figure S3d). With a water-immersion objective, and the average photon number of 374, the localization uncertainties in the x-, y-, and z-direction were 36.4 nm, 31.6 nm, and 84.4 nm, respectively. With increase average photon number (2138), the localization uncertainty in the z-direction was improved to 34.7 nm (Additional file 1: Figure S3 h).

### Whole-cell microtubule imaging

The whole cell, 3D, superresolution imaging capability of the microscope was tested using COS-7 cells. Microtubules were immunostained with biotinylated β-tubulin antibody, and then the antibodies were conjugated with biotin-modified docking strands (Additional file 1: Supplementary Table S1, docking P1) using streptavidin-biotin interaction. After completely washing out unbound biotins, streptavidins, and docking DNA strands, the imager strand (Additional file 1: Supplementary Table S1, imager P1) was injected into the detection chamber. Superresolution images of the microtubule of the whole COS-7 cell were taken at constant excitation power by moving the sample by 300 nm distance in z-direction from the glass surface to z = 6.2 μm.

**Fig. 2:**
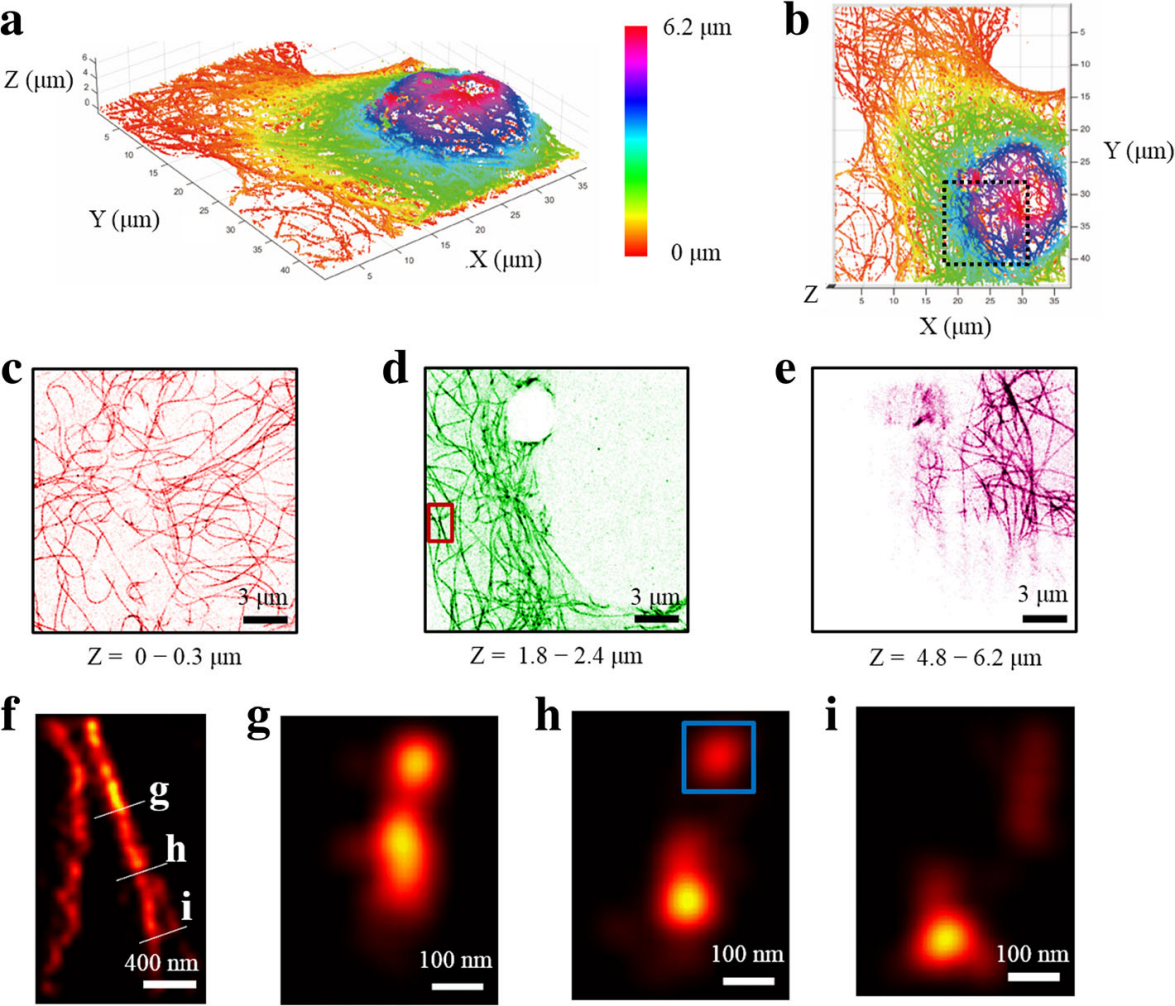
Superresolution, 3D reconstruction of microtubules of a whole COS-7 cell. **a** Perspective view of the whole-cell super-resolution image. **b** Top-view image of (**a**). **c-e** Maximum intensity projection images of black-dashed box in (**b**) at indicated z-regions. **f** Zoom-in image of the red box in (**d**). **g-i** Cross sectional images at three different positions in (**f**). In the images of **g-i**, the z-direction is aligned to the vertical direction

Figure 2a shows a perspective view of the whole-cell super-resolution image obtained by merging 3D superresolution images taken at different z-positions. In the figure, the z-position was color-coded as well. Figure 2b is a top-view image of Fig. 2a. Figure 2c-e show the maximum intensity projection images of black-dashed box in Fig. 2b for three different z-regions indicated. It is clear that microtubules wrap the nucleus at increasing z-positions whereas they were more or less evenly distributed at the bottom. Figure 2f shows the zoom-in image of the red box in Fig. 2d. Figure 2g-i show the cross sectional images in three different positions of Fig. 2f. Two diverging microtubules were clearly visualized.

In conventional superresolution fluorescence microscopy based on single-molecule localization, HILO microscopy is used. We compared the image quality of the confocal microscopy with that of HILO microscopy at varying imager concentrations. At 3 μm z-position, the signal-to-noise ratio of the line-scan confocal microscopy was better than that of HILO microscopy due to reduced background noise (Additional file 1: Figure S4). HILO microscopy could not be used at imager concentration higher than 2 nM whereas line-scan confocal microscope could be used even at 10 nM imager concentration. At z-positions higher than 10 μm, HILO microscopy could not be used for single-molecule imaging.

We characterized how the localization precision in the xy-plane varies with z-position. The average detected photon number of single-molecule spots at the bottom (Fig. 2c) was 2883, and thus the localization precision in the xy-plane was estimated as 6.14 nm. The localization precision in the xy-plane became worse with increasing z-position (Additional file 1: Figure S5a) due to the aberration of the oil-immersion objective [24]. To evaluate the resolution, we analyzed the microtubule image of Fig. 2d with the Fourier Ring Correlation [34, 35], and obtained the image resolution of 48.6 nm (Additional file 1: Figure S6). This value is comparable to the value previously reported [34].

To characterize the effect of the asymmetric localization uncertainties in the x/y- and z-direction on the image quality, we evaluated the full width at half maximum (FWHM) of microtubule image in the blue box of Fig. 2h. The FWHM of microtubule in the lateral direction is 55.6 nm whereas that in the z-direction is 85.2 nm (Additional file 1: Figure S7).

### Two-color 3D imaging

**Fig. 3:**
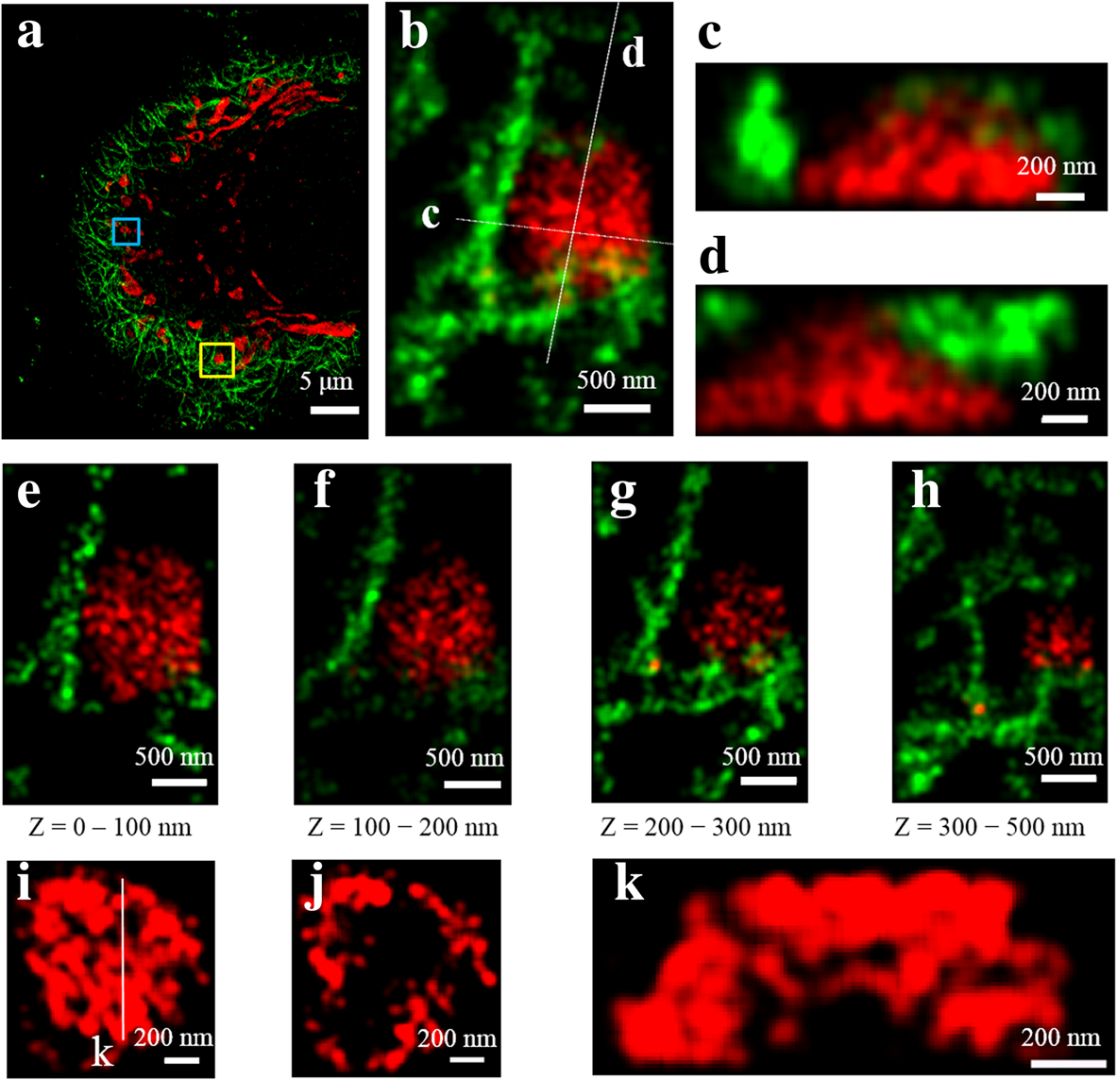
Multi-color imaging of microtubule and mitochondria. **a** Two-color super-resolution images of microtubule (green) and mitochondria (red). The image was acquired from z = 0–500 nm. **b** Zoom-in image of the yellow box in (**a**). **c-d** Cross-sectional images for white lines of (**b**). **e-h** Maximum intensity projection images for different z-regions of (**b**). **i** Zoom-in image of mitochondria of the blue box in (**a**). **j** Optical z-axis sectional image in the range of z = 0 nm to 150 nm in (**i**). **k** Cross-sectional image for white line of (**i**). In the images of (**c**), (**d**), and (**k**), the z-direction is aligned to the vertical direction

We also tested the multi-color imaging capability of the microscope. Microtubules and mitochondria were stained with docking P1 and docking P2, respectively (Additional file 1: Supplementary Table S1). Microtubules and mitochondria were sequentially imaged by first injecting imager P1 (Additional file 1: Supplementary Table S1), and next imager P2 (Additional file 1: Supplementary Table S1). The same procedure were repeated three times by increasing z-position by 200 nm. Figure 3a shows the maximum intensity projection of the merged images of microtubule (green) and mitochondria (red). Figure 3b shows the zoom-in image of the yellow box in Fig. 3a. Figure 3c-d show the cross-sectional images for white lines of Fig. 3b. Figure 3e-h show the maximum intensity projection images for different z-regions indicated. The spatial relationship between microtubules and mitochondria is clearly visualized. Figure 3i shows the zoom-in image of mitochondria of the blue box in Fig. 3a. Figure 3j describes the optical z-direction sectional image from 0 nm to 150 nm in Fig. 3i, and Fig. 3k is the cross-sectional image for the white line of Fig. 3i. The hollow structure of mitochondria is clearly visualized.

### Synapse imaging of a mouse brain tissue

We tested how the microscope operates in thick tissue samples using a section of the hippocampus region of mouse brain. We prepared 100 μm thick mouse brain tissues (Methods). The presynaptic protein, Bassoon, was stained with docking P2 whereas the postsynaptic protein, Homer1, was stained with docking P3 (Additional file 1: Supplementary Table S1). Before imaging, we injected both imager P2 labeled with Cy3B and imager P3 labeled with Alexa Fluor 647 (Additional file 1: Supplementary Table S1), and alternatively imaged Homer1 and Bassoon. The same procedure was repeated for different z-positions.

**Fig. 4:**
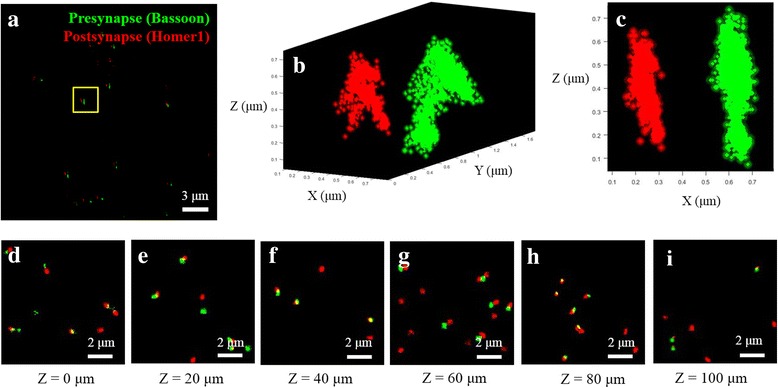
Superresolution imaging of thick brain tissue. **a** Maximum intensity projection superresolution image of presynapse (green) and postsynapse (red) of a 100 μm-thick mouse brain tissue. The image was acquired from z = 18.0 ~ 18.6 μm. **b** Perspective view of the yellow box in (**a**). **c** Side-view of the yellow box in (**a**). **d-i** Superresolution synapse images at different z-positions indicated

Figure 4a shows the presynaptic (green) and postsynaptic images (red) in the region of z = 18.0 ~ 18.6 μm. Figure 4b shows the perspective view of the yellow box region of Fig. 4a. Figure 4c shows the side-view of the same region. It is apparent that the microscope can clearly visualize the synaptic gap of thick tissue samples. The gap size was in the range of 250 nm to 300 nm, which is similar to the previously reported values [10, 36, 37]. We compared the image qualities at different imaging depths. Figure 4d-i show images in the range of 0 to 100 μm. By adjusting the illumination condition for each imaging depth, similar image qualities could be obtained. When the constant illumination condition was assumed, however, the localization precision in the xy-plane became worse from 14.0 nm at 0 μm to 30.5 nm at 100 μm (Additional file 1: Figure S5b). It was reported that the aberration of the water-immersion objective was negligible in this imaging range [24]. Therefore, we think that the deteriorated image quality was due to light scattering in the tissue [38].

## Discussion

In this study, we developed superresolution fluorescence microscopy that can reconstruct 3D structure of thick samples. It was possible by synergistically combining line-scan confocal microscopy with DNA-PAINT; while DNA-PAINT helped line-scan confocal microscopy to overcome the photobleaching problem of fluorescent probes, line-scan confocal microscopy provided the optical sectioning capability that saves DNA-PAINT method from its huge background noise.

We demonstrated that the new microscope can be used to reconstruct whole 3D structures of specimens with thickness up to 100 μm. Considering that the imaging depth of superresolution fluorescence microscopy based on single-molecule localization has been just a few micrometers, this is a significant improvement which is expected to open a way to wide application of superresolution fluorescence microscopy techniques to tissue imaging. For instance, serial section electron microscopy has been the main method that is currently available for imaging the neural connectivity with high resolution. This technique, however, is too laborious and error-prone. It does not provide clear pictures of gap junctions, and cannot distinguish whether a chemical synapse is excitatory or inhibitory. Furthermore, it takes huge amount of time to reconstruct a three-dimensional neural connection map from two-dimensional gray-scale image stacks of electron micrographs. Superresolution fluorescence microscopy has a potential to solve all of these problems, but its application to connectomics has been limited [13]. We expect our technique will change the situation, and provide a means to monitor morphological changes of synapsis during learning and memory [39].

DNA-PAINT is free from the photobleaching problem, and provides superior localization accuracy. However, we note that the slow imaging speed is an unpleasant demerit of the technique. Recently, a new technique was reported that overcomes the slow imaging speed problem of DNA-PAINT [40, 41]. The technique called FRET-PAINT improved the imaging speed of DNA-PAINT by more than 40 times, and further improvement is still expected by adopting short DNA probes and a higher speed camera. We expect a successful combination of FRET-PAINT and confocal microscopy will make the use of superresolution fluorescence microscopy for tissue imaging a routine process.

## Methods

### Optical setup

A line scan confocal microscope was built upon a commercial inverted microscope (IX71, Olympus). The backport was used to deliver line-focused 637 nm laser (Cube640-100C, Coherent), 639 nm laser (MRL-FN-639-1 W, CNI), and 532 nm laser (Compass215M, Coherent) into the microscope as well as to send fluorescence signal from a sample to an EMCCD camera (ProEM, Princeton Instruments). One galvanometric mirror (GM1 in Fig. 1, VM1000, Cambridge Technology) is inserted in the beam path and vertically scans the line-focused illumination across the sample plane. When the fluorescence signal comes back and is reflected by the same galvanometric mirror, the scanning is undone and thus the line-shaped fluorescence image is projected on a confocal slit (S50R, Thorlabs) without any vertical motion from the scanning process. The fluorescence signal passing the slit is focused on an EM-CCD camera and scanned synchronously with GM1 by using another galvanometric mirror (GM2 in Fig. 1) so that a 2D image is directly re-created on the surface of the camera. For z-positioning, a cylindrical lens (f = 400 or 1000 mm) was placed in front of the EM-CCD camera to introduce optical astigmatism [8] (Additional file 1: Figure S8). For correction of sample drift, a bright field imaging system was used. IR lamp (U-LH100, Olympus and FEL0750, Thorlabs) illuminated the sample from the top and a dichroic mirror (ZT740, Chroma) added in the read-out path separated the IR image from the fluorescence signal. The IR image was detected by an analog CCD camera connected to a computer (computer 3 in Fig. 1) via a frame grabber (PCI-1410, National Instruments) for real-time drift calculations. For cell imaging, an oil immersion objective (N.A. = 1.4, UPLSAPO100XO, Olympus) was used. For mouse brain tissue imaging, a water immersion objective (N.A. = 1.2, UPLSAPO60XW, Olympus) was used.

### Image data acquisition

Just before starting imaging, imaging buffer (5% glucose, 500 mM NaCl, 0.8 mg/mL glucose oxidase, 40 μg/mL catalase, 10 mM Tris-HCl (pH 8.0) in saturated Trolox solution) containing imager strands (0.2–1 nM) was injected into the channel. DNA-PAINT images were typically recorded at a frame rate of 3.3 Hz while scanning galvanometric mirrors at 50 Hz. This multi-scan-per-frame scheme helps the images of free fluorophores in the solution appear uniformly, since they moved in and out of the focal plane during the exposure time for one frame. Imaging per one focal plane typically took one to two hours (10,000–20,000 frames). Imaging buffer was exchanged every hour using a syringe pump (Fusion 200, Chemyx Inc.). For drift correction in real-time, we took one in-focus and two out-out-focus bright-field images just before starting imaging as references to keep track of the x-, y-, and z-drifts of the sample during the imaging period. In case of tissue imaging, bright field image of tissue itself provided good enough contrast for drift correction, but in case of cell imaging, the presence of the latex bead was critical for drift correction [8]. During image recording, the amounts of drift in the x-, y-, and z-direction were calculated and recorded by the computer 3. The drift in the z-direction was corrected in real-time. The drifts in the x-, y-direction were checked at every 900 s, and corrected if the amount of the drifts were larger than the threshold value (60 nm) using the translation stage (PZ-2000, Applied Scientific Instrumentation) (Additional file 1: Figure S9a-b). Additional file 1: Figure S9c-d showed that our drift correction system works well.

### Image processing

The raw image data were first processed by a mean filter to remove high frequency noise and then searched for all local maxima above a certain threshold. 13 × 13 pixel raw images around these maximum points were recorded, and the ones that appeared consecutively in the same location with displacements less than 3 pixels were combined to make whole single-molecule images. They were then fitted to a 2D elliptical Gaussian function to extract the height, center position, and x/y-widths of the Gaussian function. The total image intensity, the number of frames, and the deviation from the Gaussian fit were used to filter out unreliable data. Based on the drift data recorded, the center position of the single-molecule spots was corrected as shown in Additional file 1: Figure S10. Additional file 1: Figure S11a-b show x/y-widths of surface immobilized single-molecules at varying z-positions with a 100 nm step. To obtain a z-calibration curve based on these data, the average x/y-width of surface-immobilized spots were calculated for each z-position, and a calibration curve was produced in the two-dimensional plane of x-width and y-width by connecting these averaged points (Additional file 1: Figure S11c). To assign the z-position to individual single-molecule spots of samples, the point of the spot in the plane of x-width and y-width was projected to the nearest z-calibration line, and the relative position of the projection was used to determine the z-position of each spot (Additional file 1: Figure S11c). Based on these calibration data, each single-molecule was assigned a z-offset value from the focal plane, and the overall z-position was rescaled by a factor of 0.79 to correct for the difference between the actual and apparent focal plane due to the refractive index mismatch [9]. To obtain good z-localization accuracy, we avoided the z-regions that significantly overlap in Additional file 1: Figure S11a-b, and used for 3D image reconstruction only the z-assignment in the range from − 100 nm to + 200 nm or 0 to + 200 nm. For Fig. 2, each localization was represented as a point without any Gaussian blur, which allowed fast rendering while we rotated it in 3D. For rendering of the superresolution images presented in Figs. 3 and 4, a Gaussian function of 30 nm width was used to represent each localization. To prevent false localizations in the background from obscuring the view, data shown in Figs. 3 and 4 were further processed using Image Segmenter of MATLAB software (MathWorks) so that only the points forming dense clusters remained while loosely scattered points were considered as noise and discarded.

### GPU computing

For fast image processing, we used an independent computer equipped with 4 GPUs (GTX780, Nvidia). Use of Parallel Computing Toolbox of MATLAB software (MathWorks) allowed us to take advantage of parallel computing power of all four GPUs with no special training on GPU programming languages such as CUDA. When our main computer received raw images from the EM-CCD camera, every 500 frames were grouped together and saved as a separate file. Each file was then loaded by the GPU computer through the network and then processed via one of the available GPUs while the image recording still took place in the main computer. Even without a serious optimization, our image processing system could keep up with a stream of 512 × 512 image data coming at 20 Hz or faster.

## Materials

Modified DNA oligonucleotides were purchased from Integrated DNA Technologies. Biotinylated β-tubulin antibody (catalog number: S6181) were purchased from Cell Signaling. Biotinylated anti-mitochondria antibody (catalog number: ab79479) and anti-tubulin antibody (catalog number: ab6160) were purchased from Abcam. Tom20 antibody (catalog number: sc-11415) was purchased from Santa Cruz Biotechnology, Inc. Anti-Bassoon antibody (catalog number: 141003) and anti-Homer1 antibody (catalog number: 160011) were purchased from Synaptic Systems. Donkey anti-rabbit IgG antibody (catalog number: 711–005-152), donkey anti-rat IgG antibody (catalog number: 712–005-153), and donkey anti-mouse IgG antibody (catalog number: 715–005-151) were purchased from Jackson ImmunoResearch Laboratories Inc. Streptavidin (catalog number: S-888), carboxyl latex beads (catalog number: C37281), phosphate-buffered saline 10× (catalog number: 70011–044), Dulbecco’s modified Eagle’s medium (catalog number: 11995065), Fetal Bovine Serum (catalog number: 16000044), and Penicillin-Streptomycin (catalog number: 15140122) were purchased from Thermo Fisher Scientific. Normal donkey serum (catalog number: 017–000-121) was purchased from Jackson ImmunoResearch Laboratories Inc. Antibody-oligonucleotide all-in-one conjugation kit (catalog number: A-9202-001) was purchased from Solulink. Paraformaldehyde (catalog number: 1.04005.1000) was purchased from Merck. Glutaraldehyde (catalog number: G5882), Triton X-100 (catalog number: T9284), and Poly-L-lysine (catalog number: P8920) were purchased from Sigma-Aldrich.

### Cell preparation

The COS-7 cells were purchased from Korean Cell Line Bank. The COS-7 cells of 20–30 passages were grown in a cell culture flask containing Dulbecco’s modified Eagle’s medium with 10% fetal bovine serum and 1% penicillin-streptomycin at 37 °C. The level of CO_2_ was maintained at 5% during the cell culture.

For drift correction of cell imaging, #1.5 glass coverslips were sparsely coated with carboxyl latex beads. The coverslip was coated with bead solution 1:10 diluted in distilled water, heated for 10 min on a 100 °C hot plate, washed thoroughly with distilled water, and dried with N_2_ gas. COS-7 cells were grown on bead-coated coverslips for a few days and then fixed with the mixture of 3% paraformaldehyde and 0.1% glutaraldehyde in PBS buffer for 10 min and stored at 4 °C in PBS buffer until needed. A flow channel was made by assembling the cell-covered coverslip and a glass slide using double-sided tape and epoxy [42]. In the glass slide, two holes were pre-made for convenient buffer exchange.

For microtubule imaging (Fig. 2), microtubules were immunostained by injecting 10 μg/mL biotinylated β-tubulin antibody mixed in 30 μL blocking solution (3% Normal Donkey Serum and 0.25% Triton X-100 in PBS) into the channel and incubating for 1 h. 0.2 mg/mL streptavidin in the blocking solution, and 500 nM biotinylated docking P1 in the blocking solution were sequentially injected with 5 min incubation, and thorough PBS washes between each step.

For two-color imaging of microtubules and mitochondria (Fig. 3), microtubules were immunostained by injecting 1:200 diluted anti-tubulin antibody in blocking solution into the channel and incubating at 4 °C overnight. After thorough wash-out of free anti-tubulins with PBS buffer, cells were incubated with 50 nM secondary antibody conjugated with docking strand (Additional file 1: Supplementary Table S1, docking P1) for 1 h. Then, mitochondria were immunostained by injecting 10 μg/mL biotinylated anti-mitochondria antibody mixed in 30 μL blocking solution into the channel and incubating for 1 h. 0.2 mg/mL streptavidin in the blocking solution, and 500 nM biotinylated docking P2 in the blocking solution were sequentially injected with 5 min incubation, and thorough PBS washes between each step.

### Mouse brain tissue preparation

Adult C57BL6/N mice (10~ 16 weeks) from KOATAK were anesthetized with ketamine/xylazine and whole body was fixed with perfusion with 4% PFA in PBS. After perfusion, brain was extracted and kept at 4 °C for overnight. From next day, fixed brains were transferred consecutively from 15% to 30% sucrose solution at 4 °C for overnight. Mice brains were frozen in Tissue-Tek O.C.T. compound block at − 80 °C for overnight. The 100 μm coronal brain slices were made with Leica CS3050S and kept in 50% Glycerol in PBS at − 20 °C.

Fixed mouse brain tissues were immunostained in a mixture of 1:100 diluted anti-Bassoon antibody (for presynapses) and 1:100 diluted anti-Homer1 antibody (for postsynapses) in blocking solution in a 24 well plate at 4 °C overnight. After thorough wash-out of free antibodies with PBS buffer for 20 min, mouse brain tissues were incubated with 100 nM donkey anti-rabbit antibody conjugated with docking P2 (Additional file 1: Supplementary Table S1) and donkey anti-mouse antibody conjugated with docking P3 (Additional file 1: Supplementary Table S1) at 4 °C overnight. After thorough wash-out of free secondary antibodies with PBS buffer for 20 min, mouse brain tissues were mounted on #1.5 glass coverslip coated by Poly-L-lysine. A flow channel was made by assembling the tissue-mounted coverslip and a glass slide with two holes for buffer exchange using double-sided tape and epoxy [42].

All experimental protocols were approved by the Seoul National University Institutional Animal Care and Use Committee and were in accordance with guidelines from the Seoul National University Institutional Biosafety Committee.

## Additional file

Additional file 1: Superresolution fluorescence microscopy for 3D reconstruction of thick samples. (PDF 914 kb)
