# Supplementary material for: Superresolution fluorescence microscopy for 3D reconstruction of thick samples
Source: Mol Brain. 2018 Mar 15;11:17. doi: 10.1186/s13041-018-0361-z (PMC5856285; doi:10.1186/s13041-018-0361-z)
Supplement: Supplementary file 1 — Superresolution fluorescence microscopy for 3D reconstruction of thick samples. (PDF 914 kb) [file 13041_2018_361_MOESM1_ESM.pdf]

Additional file 1

Superresolution fluorescence microscopy for 3D reconstruction of thick samples

Sangjun Park[1-3,6], Wooyoung Kang[1-3,6], Yeong-Dae Kwon[3,4], Jaehoon Shim[5], Siyong Kim[5], Bong-Kiun Kaang[5], and Sungchul Hohng[1-3, *]

Institutional address:

[1]Department of Physics and Astronomy, Seoul National University, Seoul, Republic of Korea.

[2]Institute of Applied Physics, Seoul National University, Seoul, Republic of Korea.

[3]National Center of Creative Research Initiatives, Seoul National University, Seoul, Republic of Korea.

[4]Research Institute of Basic Sciences, Seoul National University, Seoul, Republic of Korea.

[5]School of Biological Sciences, Seoul National University, Seoul, Korea.

[6]These authors contributed equally to this work.

E-mail address:

Sangjun Park: cygnus30@snu.ac.kr

Wooyoung Kang: crn1860@gmail.com

Yeong-Dae Kwon: yeongdae@gmail.com

Jaehoon Shim: jhshim11@snu.ac.kr

Siyong Kim: siyongkim@snu.ac.kr

Bong-Kiun Kaang: kaang@snu.ac.kr

Sungchul Hohng: shohng@snu.ac.kr

* Correspondence should be addressed to SH (shohng@snu.ac.kr).

**Supplementary Figure S1.**	Influence of the slit insertion on the detected photon number
**Supplementary Figure S2.**	Variation of x- and y-widths at different z-positions
**Supplementary Figure S3.**	Characterization of localization uncertainties of the microscope
**Supplementary Figure S4.**	Comparison of signal-to-noise ratios of line-scan confocal microscopy and HILO microscopy
**Supplementary Figure S5.**	Localization precision as a function of imaging depth
**Supplementary Figure S6.**	Fourier Ring Correlation analysis of Figure 2d
**Supplementary Figure S7.**	Asymmetry of microtubule images
**Supplementary Figure S8.**	Raw data images of the microscope
**Supplementary Figure S9.**	Drift correction method
**Supplementary Figure S10.**	Drift correction during post-imaging analysis
**Supplementary Figure S11.**	Assignment of the z-position of single-molecule spots
**Supplementary Table S1.**	Docking and imager strand sequences for microtubule, mitochondria, presynapse, and postsynapse imaging.

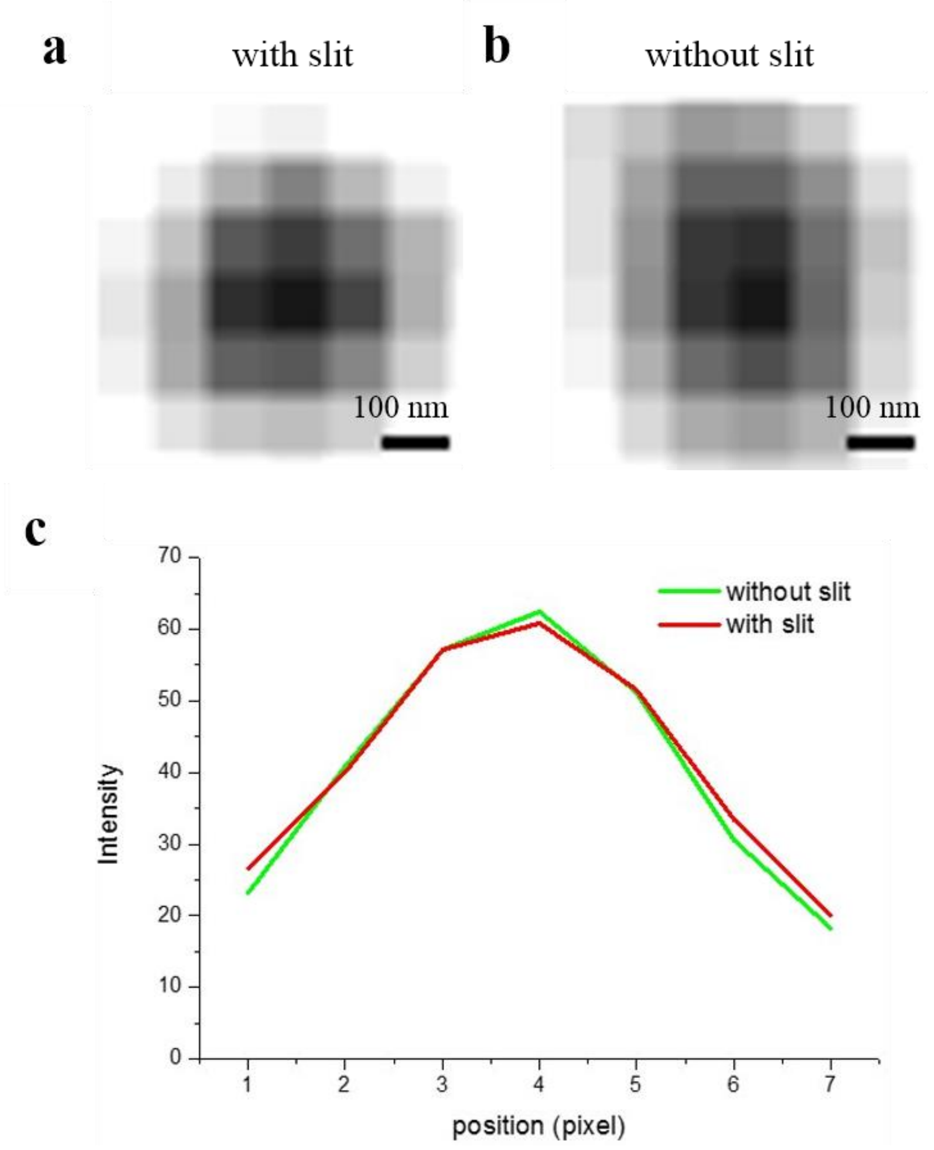

Additional file 1: Supplementary Figure S1. Influence of the slit insertion on the detected photon number. The slit width was 50 μm. **a** Single-molecule image with a slit. **b** Single-molecule image without a slit. **c** Integrated intensity of (**a**) (red lines) and (**b**) (green lines) projected along the vertical direction. The total photon number of (**a**) was 96.8% of that of (b).

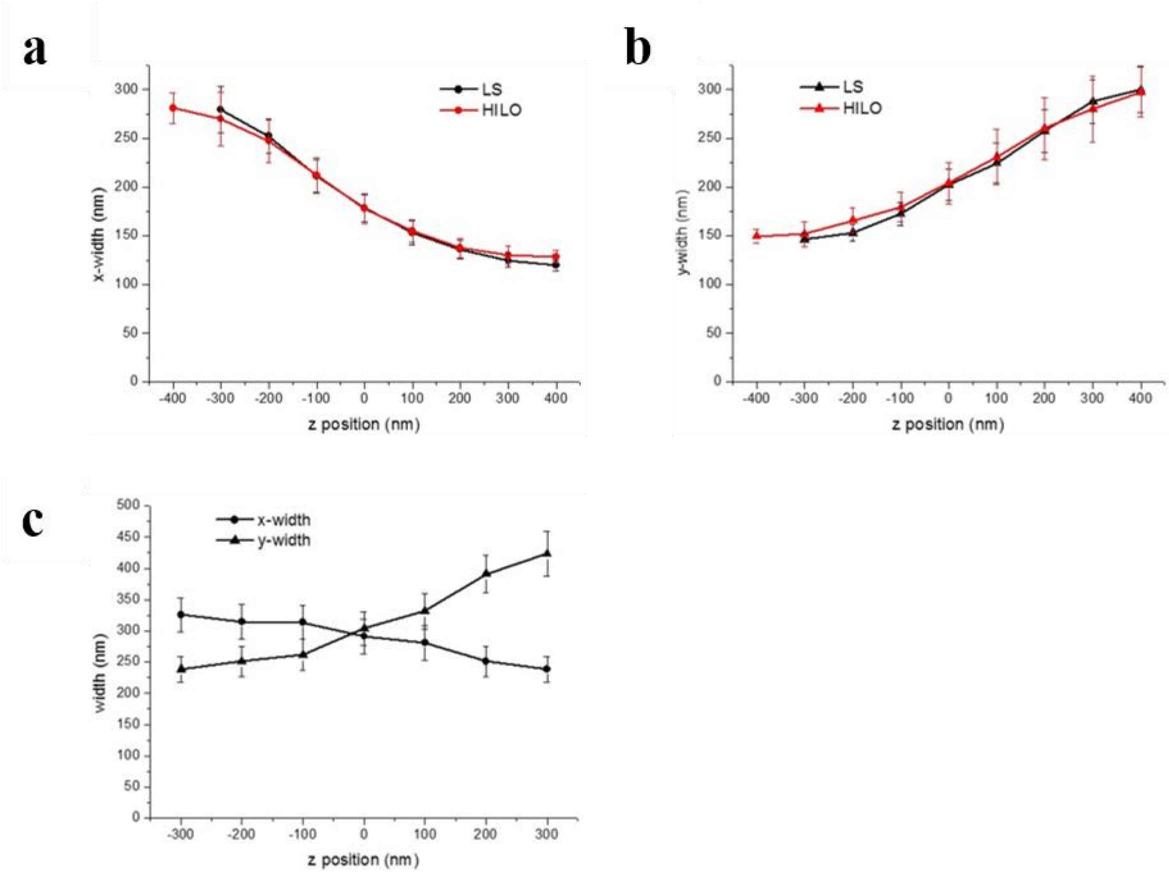

Additional file 1: Supplementary Figure S2. Variation of x- and y-widths at different z-positions. Surface-immobilized single-molecules were imaged by moving the sample with a 100 nm step, and fitted to 2D elliptical Gaussian functions to obtain x- and y-widths. **a-b** x-width and y-width variations in a line-scan confocal microscope (black) and a HILO microscope (red). An oil-immersion objective was used. At z = -400 nm, single-molecules were not detected in a line-scan confocal microscope. **c** x-width (circle) and y-width (triangle) variations in a line-scan confocal microscope with a water-immersion objective.

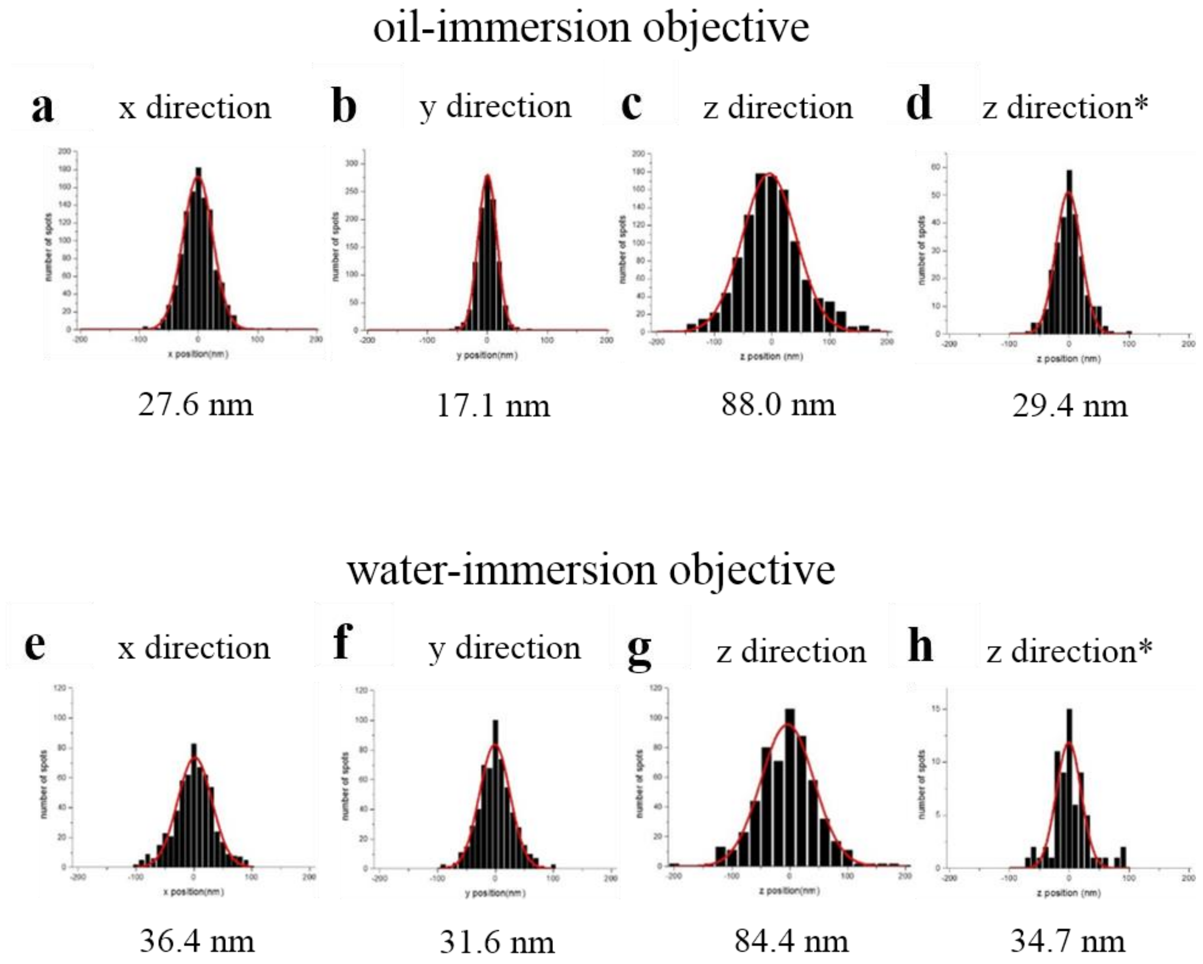

Additional file 1: Supplementary Figure S3. Characterization of localization uncertainties of the microscope. **a-c** Localized position distributions of surface-immobilized single-molecules obtained with an oil-immersion objective when the average photon number was 623. The localization uncertainties defined as the standard deviation of the distribution are presented below the figures. With an increased average photon number from 623 to 3,730, the localization uncertainty in the z-direction was improved to 29.4 nm (**d**). **e-g** Localized position distributions of surface-immobilized single-molecules were obtained with a water-immersion objective when the average photon number was 374. The localization uncertainties defined as the standard deviation of the distribution were presented below the figures. With an increased average photon number from 374 to 2,138, the localization uncertainty in the z-direction was improved to 34.7 nm (**h**).

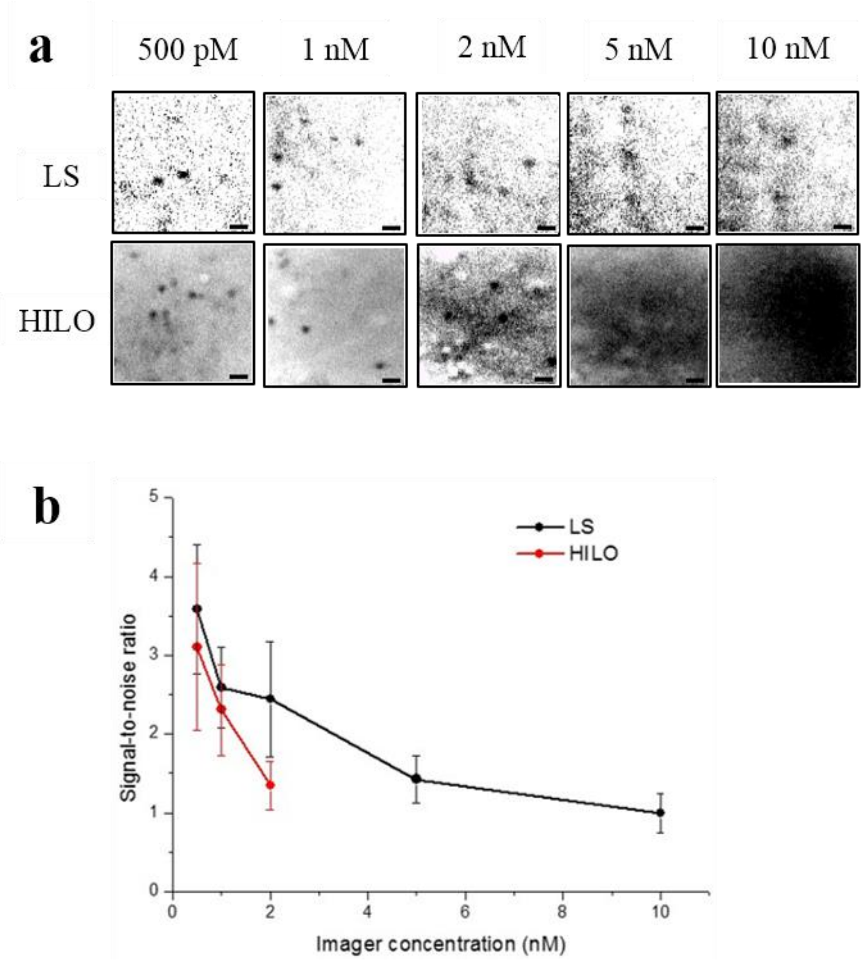

Additional file 1: Supplementary Figure S4. Comparison of signal-to-noise ratios of line-scan confocal microscopy and HILO microscopy. Data obtained to acquire microtubule images as shown in Fig. 2 were used for the comparison. **a** Single-molecule images in the line-scan confocal microscope (LS) and those in the HILO microscope (HILO) at fixed z-position (3 μm) and varying imager concentrations. **b** Signal-to-noise ratios of the single-molecule images in (**a**) as a function of imager concentration. Scale bars in (**a**): 1 μm.

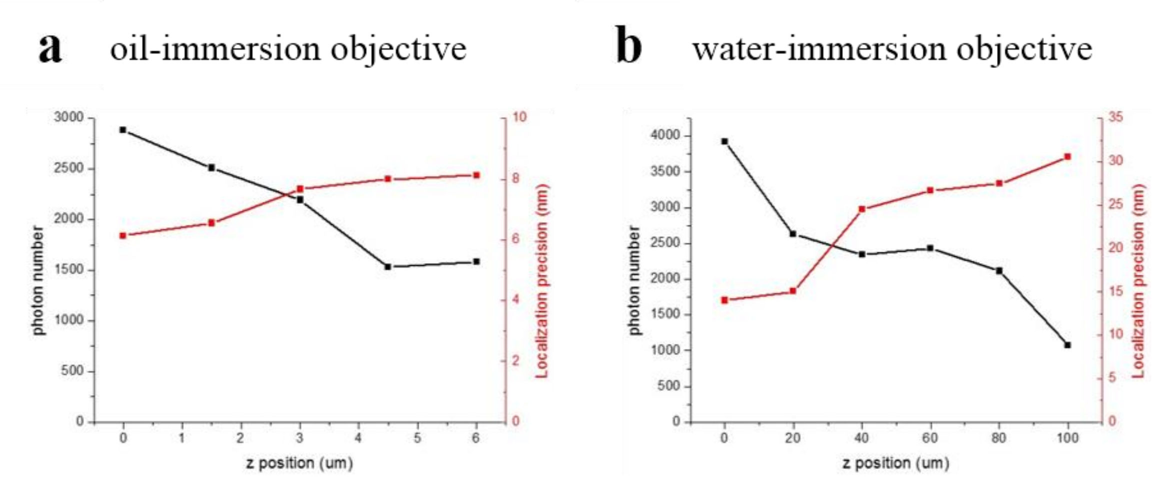

Additional file 1: Supplementary Figure S5. Localization precision as a function of imaging depth. **a** Average photon number (black) and corresponding localization precision (red) of Fig. 2 as a function of image depth. Constant illumination power was used. **b** Average photon number (black) and corresponding localization precision (red) of Fig. 4 as a function of image depth. In the experiment, the illumination condition was adjusted to obtain a uniform localization precision, but in this plot the real detected photon number was rescaled as expected when the same illumination power at the bottom was used.

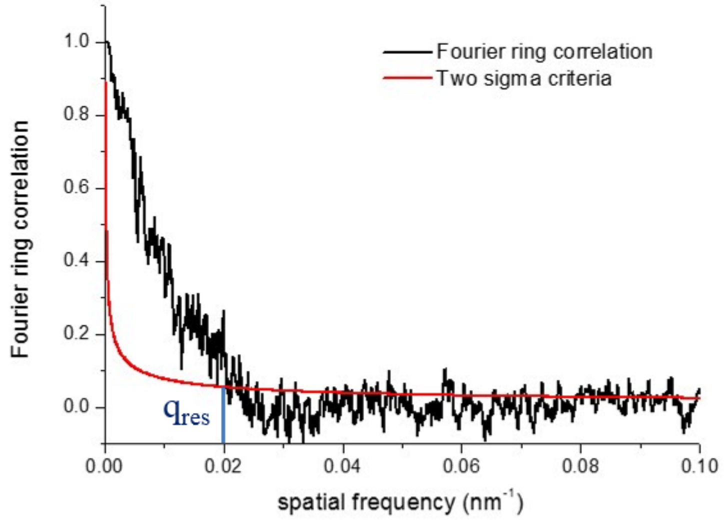

Additional file 1: Supplementary Figure S6. Fourier Ring Correlation analysis of Fig. 2d. The value of q_{res} was 0.02056 nm^{-1} and the corresponding resolution was 48.6 nm.

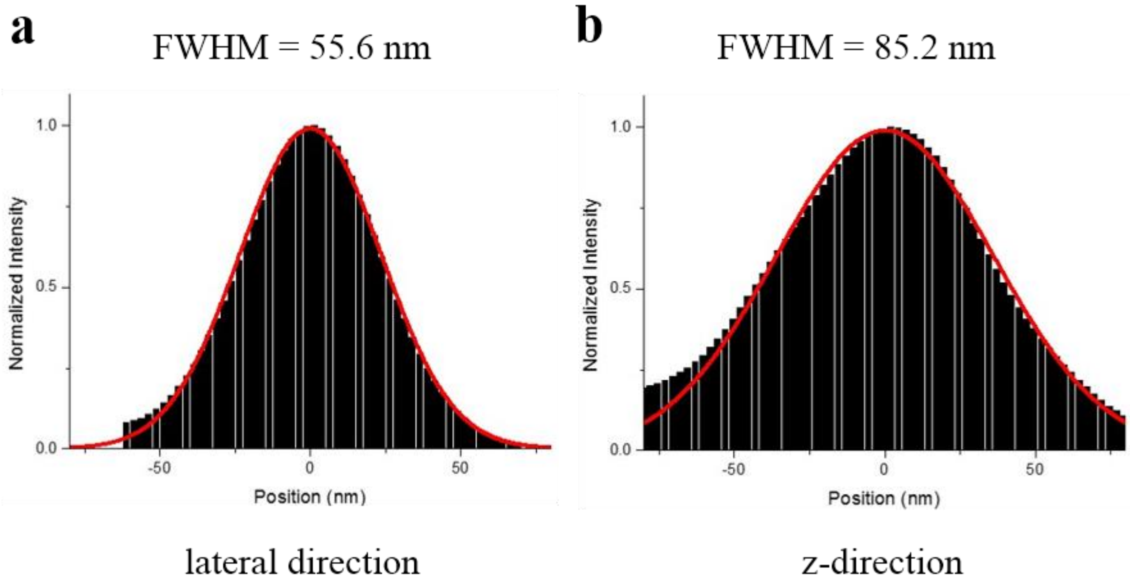

Additional file 1: Supplementary Figure S7. Asymmetry of microtubule images. The microtubule in the blue box of Fig. 2h was used for the data analysis. **a** Cross-sectional profile of the microtubule in the lateral direction. The value of FWHM was 55.6 nm. **b** Cross-sectional profile of the microtubule in the z-direction. The value of FWHM was 85.2 nm.

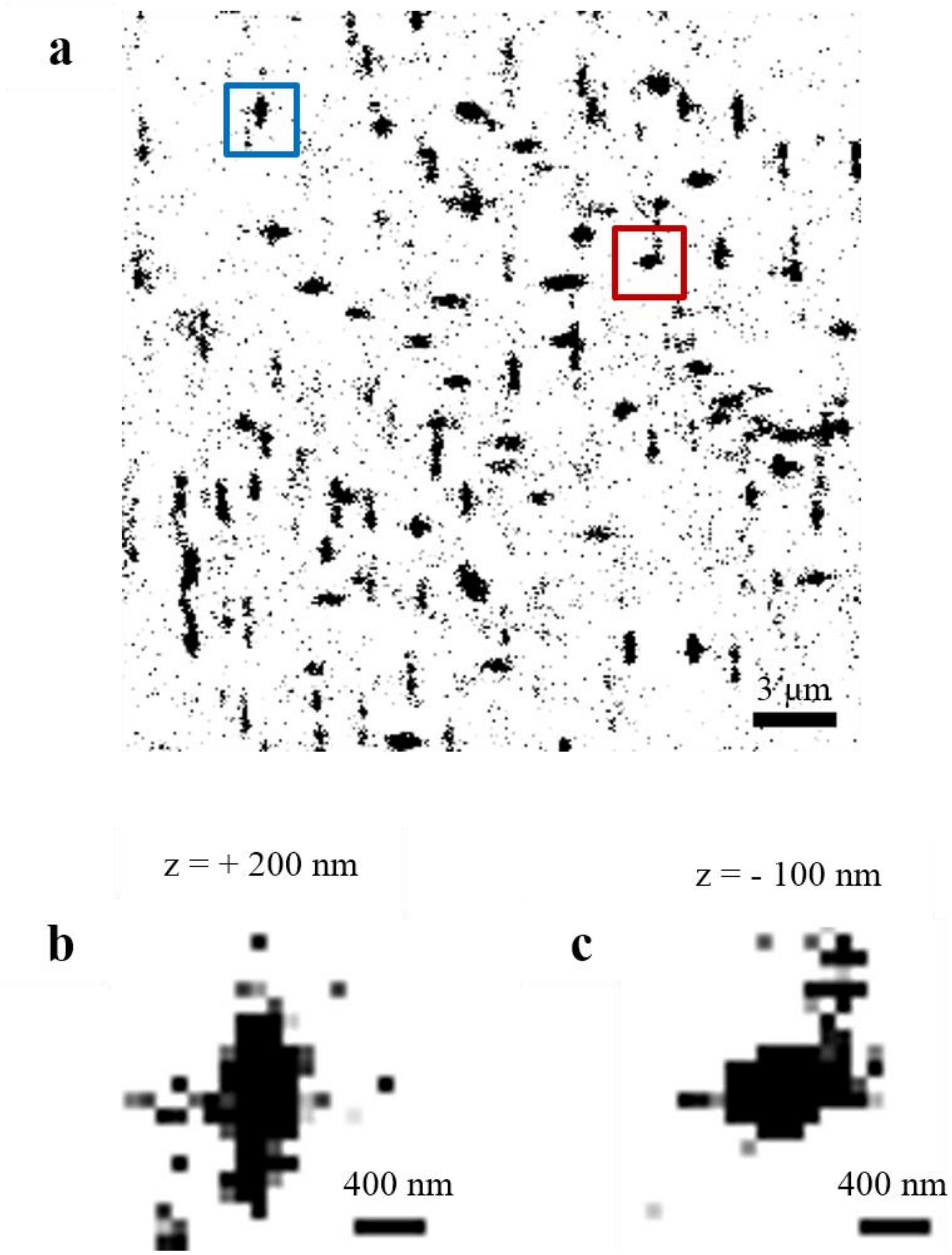

Additional file 1: Supplementary Figure S8. Raw data images of the microscope. **a** A representative raw data image that was used to reconstruct Fig. 2. **b** A zoom-in image of the blue box in (**a**). This spot was assigned at z = +200 nm. **c** A zoom-in image of the red box in (**a**). This spot was assigned at z = -100 nm.

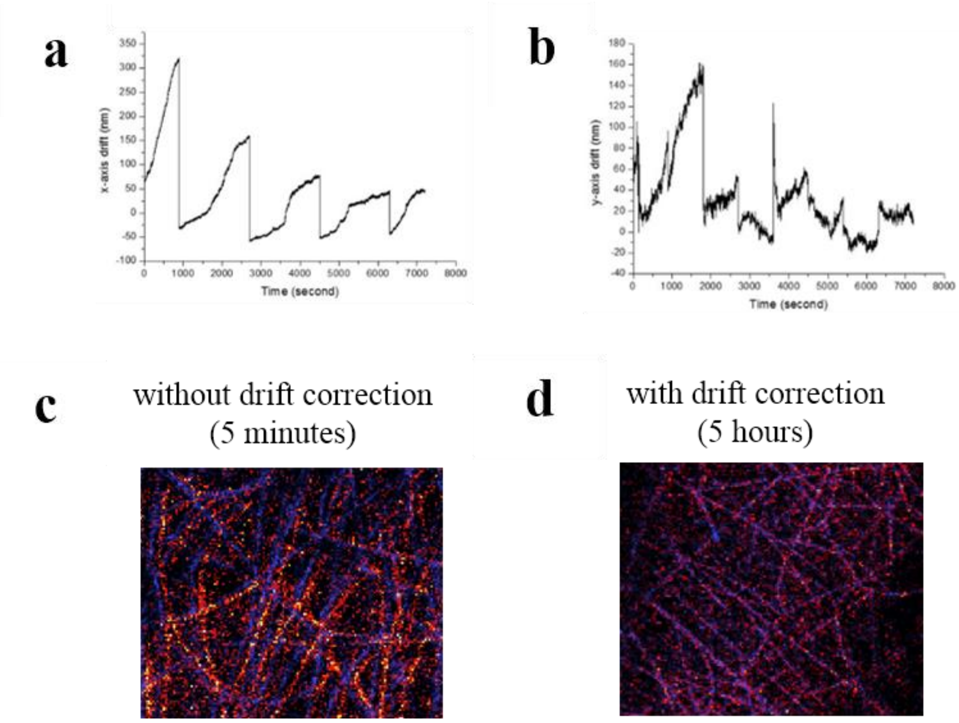

Additional file 1: Supplementary Figure S9. Drift correction method. Sample time traces of the drift in the x-direction (**a**) and the y-direction (**b**). The drifts in the x-, y-direction were checked at every 900 seconds, and corrected if the amount of the drifts were larger than the threshold value (60 nm) using the translation stage. **c** Two microtubule images consecutively obtained with 5 minute time gap during which the drift correction system was off. **d** Two microtubule images consecutively obtained with 5 hour time gap during which the drift correction system was on.

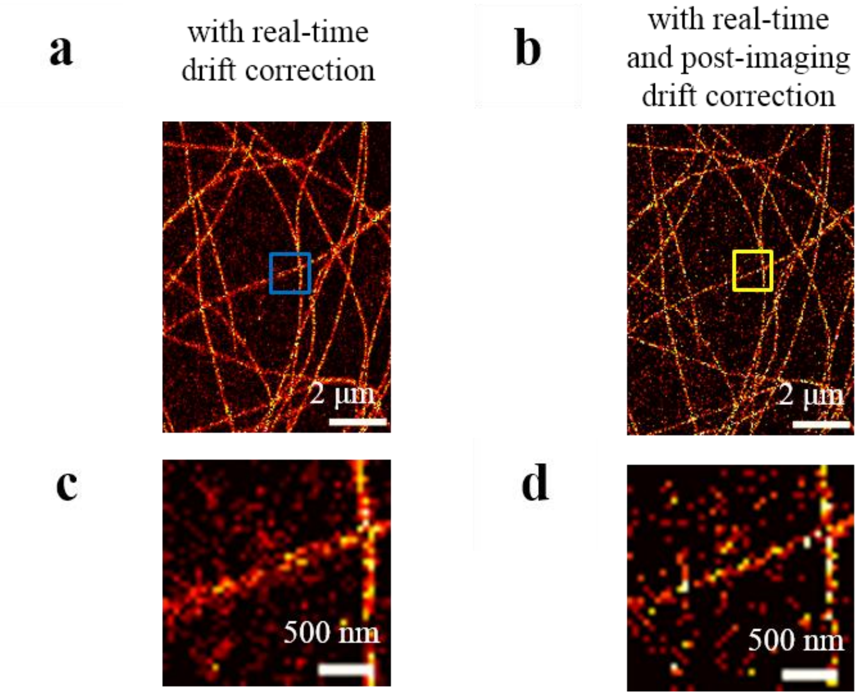

Additional file 1: Supplementary Figure S10. Drift correction during post-imaging analysis. **a** Microtubule images only with real-time drift correction. **b** Microtubule image with both real-time and post-imaging drift correction. **c** A zoom-in image of the blue box in (**a**). **d** A zoom-in image of the yellow box in (**b**).

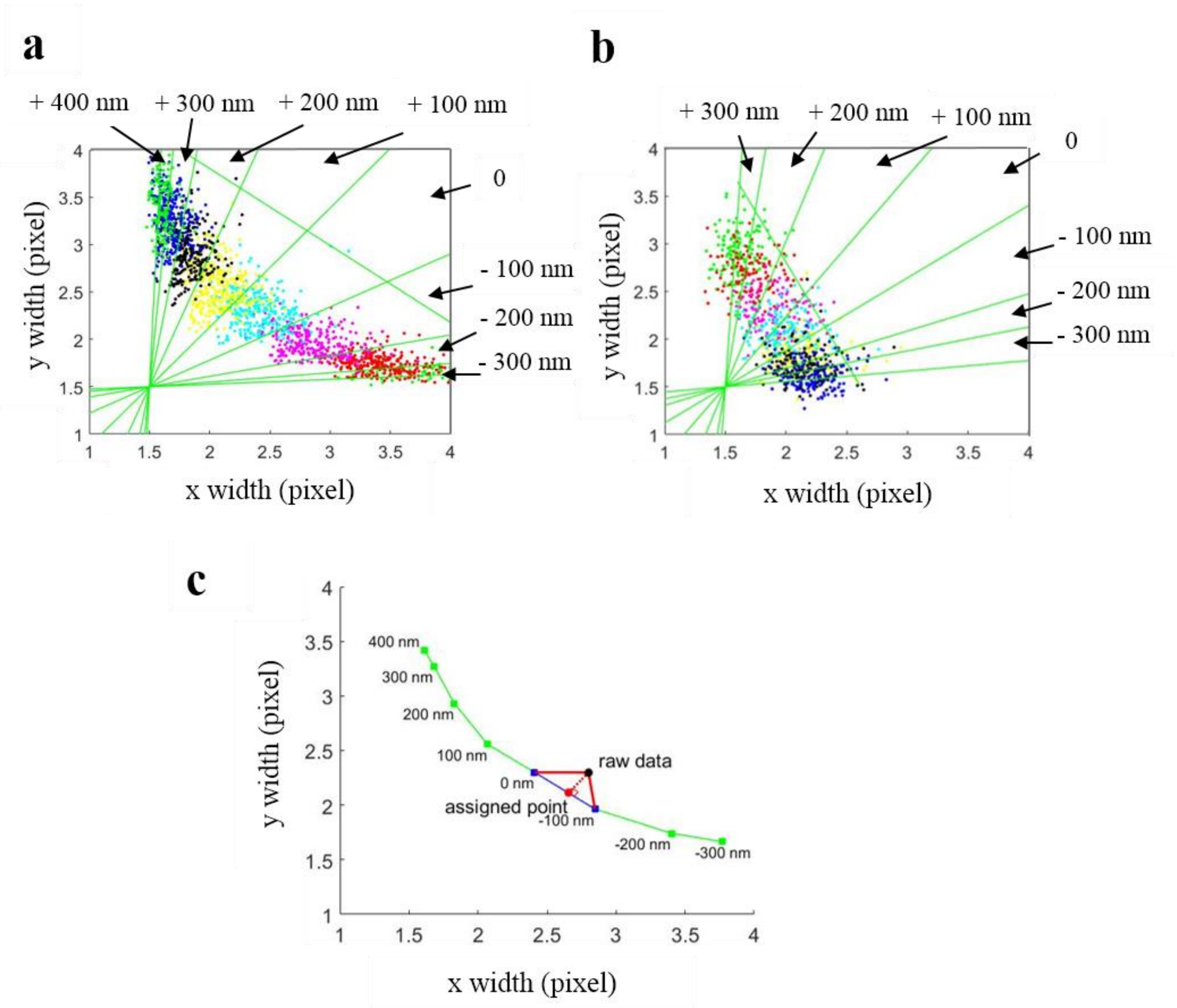

Additional file 1: Supplementary Figure S11. Assignment of the z-position of single-molecule spots. **a** Distribution of the x- and y-widths of surface-immobilized molecule images at different z-positons. The data were obtained with an oil-immersion objective and a cylindrical lens with f = 400 mm. **b** Distribution of the x- and y-widths of surface-immobilized molecule images at different z-positons. The data were obtained with a water-immersion objective and a cylindrical lens with f = 1000 mm. **c** A scheme to assign the z-position to single-molecules. The point corresponding to the single-molecule image (black circle) was projected to the nearest z-calibration line (blue) and the relative position of the projection (red circle) was used for z-assignment.

Description	Sequence
Docking P1 (Biotinylated)	5’ – Biotin – TTATACATCTAG – 3’
Docking P1 (Amine modified)	5’ – Amino Modifier – TTATACATCTAGTTTTTTTTTTTTTTTTTT – 3’
Docking P2 (Biotinylated)	5’ – Biotin – TGATCTACATA – 3’
Docking P2 (Amine modified)	5’ – Amino Modifier – TGATCTACATATTTTTTTTTTTTTTTTTTT – 3’
Docking P3 (Amine modified)	5’ – Amino Modifier – TTTCTTCATTACTTTTTTTTTTTTTTTTTT – 3’
Imager P1	5’ – CTAGATGTAT – Alexa Fluor 647 – 3’
Imager P2	5’ – TATGTAGATC – Alexa Fluor 647 – 3’<br>5’ – TATGTAGATC – Cy3B – 3’
Imager P3	5’ – GTAATGAAGA – Alexa Fluor 647 – 3’

Additional file 1: Supplementary Table S1. Docking and imager strand sequences for microtubule, mitochondria, presynapse, and postsynapse imaging.
